# Advancing Soft Tissue Reconstruction with a Ready-to-Use Human Adipose Allograft

**DOI:** 10.3390/bioengineering12060612

**Published:** 2025-06-04

**Authors:** Victor Fanniel, Ihab Atawneh, Jonathan Savoie, Michelle Izaguirre-Ramirez, Joanna Marquez, Christopher Khorsandi, Shauna Hill

**Affiliations:** 1 Shauna Hill, RegenTX Labs, LLC, 3463 Magic Dr Ste 315, San Antonio, TX 78229, USA; 2 VIP Plastic Surgery, 2779 Sunridge Heights Pkwy Ste 100, Henderson, NV 89052, USA

**Keywords:** adipose allograft, minimal manipulation, biocompatibility, soft tissue reconstruction, adipose extracellular matrix, tissue engineering

## Abstract

Soft tissue reconstruction remains a challenge in clinical practice, particularly for restoring substantial volume loss due to surgical resections or contour deformities. Current methods, such as autologous fat transplantation, have limitations, including donor site morbidity and insufficient tissue availability, necessitating an innovative approach. This study characterizes alloClae, a minimally manipulated human-derived adipose allograft prepared using a detergent-based protocol to reduce DNA content while preserving adipose tissue structure. Proteomic analysis revealed that alloClae retains key native proteins critical for graft integration with the host and stability, with key extracellular matrix (ECM) components, collagens, elastins, and laminin, which are more concentrated as a result of the detergent-based protocol. Biocompatibility of alloClae was assessed in vitro using cytotoxicity and cell viability assays in fibroblast cultures, revealing no adverse effects on cell viability, membrane integrity, or oxidative stress. Additionally, in vitro studies with adipose-derived stem cells (ASCs) demonstrated attachment and differentiation, with lipid droplet accumulation observed by day 14, indicating support for adipogenesis. A 6-month longitudinal study in athymic mice showed stable graft retention, host cell infiltration, and formation of new adipocytes and vasculature within alloClae by 3 months. The findings highlight alloClae’s ability to support host-driven adipogenesis and angiogenesis while maintaining graft stability throughout the study period. It presents a promising alternative to the existing graft materials, offering a clinically translatable solution for soft tissue reconstruction.

## 1. Introduction

Reconstruction of soft tissue, particularly for large volume tissue losses that can result from situations such as surgical resections or contour deformities, represents a significant clinical challenge. In the clinic, adipose tissue is used to replace large volumes in soft tissue reconstruction due to its rich native extracellular matrix (ECM) to facilitate tissue repair and regeneration [1,2]. The current gold-standard methods for soft tissue reconstruction involve autologous fat transplantation, which requires harvesting the patient’s own fat [3,4]. While effective, this method requires an additional surgical site, leading to donor site pain, morbidity, and, in some cases, insufficient donor fat for large volume replacement [5]. In addition, fat harvesting may also lead to soft tissue irregularities, particularly contour deformities at the donor site due to uneven fat removal or damage to adipose tissue, leading to additional surgeries or procedures and reduced quality of life [6].

Recognizing these challenges, tissue engineering plays a role in advancing reconstructive and regenerative medicine, focusing on developing grafts that simulate adipose function [1,2,7]. Adipose tissue has the potential to play a pivotal role in soft tissue engineering, as it is rich in the connective tissue matrix, provides cushioning and support for other tissues, and insulates the body [7,8,9,10]. Central to its structural function of cushioning and support are key ECM proteins, including collagens, elastins, and laminins, which also provide anchorage and barrier functions for native adipocytes, underscoring the importance of preserving these components in engineered grafts [11]. The ECM’s complex structure and biochemical composition are essential for supporting cellular functions, including adhesion, adipogenesis, and angiogenesis of host tissue for integration, which are important for regeneration [10,12,13].

Despite the potential of synthetic polymers for adipose tissue engineering, their use is often limited by the inability to adequately replace large volumes, short retention times, migration of grafts, and challenges in fabricating natural, three-dimensional constructs that reliably replicate tissue architecture and function [14,15,16]. As a result, allogenic use of adipose tissue for large volume tissue replacement is a potential avenue for clinical use. However, shifting towards clinical use of allogenic adipose tissue is challenged by complications such as capsular contracture and necrosis, underscoring the need for innovative approaches for improved techniques such as decellularization to ensure successful acceptance and tissue integration [17,18]. Decellularization techniques represent a key solution for allogenic use of adipose grafts. By reducing cellular DNA and debris from adipose tissue, this method can minimize immunogenic responses while preserving essential ECM components vital for maintaining the grafts’ utility and biocompatibility [13].

Despite the potential of decellularized adipose tissue in various clinical applications, refining decellularization to reduce processing time, while maintaining key characteristics, remains a challenge. Approaches include detergent-based and detergent-free protocols [19], both aiming to maintain the integrity of the ECM structure. Detergent-free methods, while effective in maintaining the ECM’s structural integrity and fostering an ideal microenvironment for adipose-derived stem cells (ASCs), face limitations due to their lengthy processing times, which range from 120 to 156 h, and their capacity to process no more than 800 mg of tissue at a time [19,20]. Conversely, detergent-based methods often lead to protein loss. Although they retain collagens, critical components such as laminins, vital for cell differentiation and migration, and adhesin are often reduced or absent in the final ECM [21,22]. Furthermore, these methods struggle with effectively reducing DNA and vary widely in processing times from 24 to 214 h [19,22,23]. Despite these challenges, numerous decellularized allografts have shown promise in preclinical [24,25,26] and clinical studies [27,28,29,30], underscoring the need for further refinement to enhance ECM retention, and thus support adipogenesis, angiogenesis, and long-term graft retention and improve overall outcomes [24,25,31].

Here, we characterize alloClae, a minimally manipulated adipose tissue allograft that preserves essential characteristics of adipose tissue, including adipocyte structure, ECM components, and features that support cell attachment and adipogenesis. Understanding the current clinical challenges and limitations of the current adipose decellularization techniques, the processing methods for alloClae were optimized, building on previous works. Unlike complete decellularization, our gentle, detergent-based protocol results in a product that retains adipose tissue structure while reducing genetic material to minimize the risk of host rejection. This approach not only preserves the natural bulk of adipose tissue, but also offers immediate volume, cushioning, and support. These findings were further substantiated by a 6-month mouse survival study that demonstrated alloClae’s early integration stages, adipogenesis, and angiogenesis. Our findings provide a solid foundation for the future development and application of adipose tissue allografts in clinical settings, offering promising prospects for enhancing outcomes in tissue reconstruction.

## 2. Materials and Methods

### 2.1. Production of Human-Derived Adipose Allografts

Adipose allografts were derived from 34 donated cadaveric human adipose tissues screened and recovered by FDA-registered tissue banks in accordance with the American Association of Tissue Banks (AATB) standards and FDA regulations (21 CFR Part 1271). The donors for this study included both males and females, aged 18–71 years old. The non-viable donor tissue was placed through a freeze/thaw cycle, debrided and cleared of bruised tissue, fascia, vascular tissues, and muscle. After debridement, the tissue was sent through a tissue size reduction protocol that includes a series of rinses with gentle agitation using a polyethylene glycol-based detergent and sodium chloride solutions to remove lipids and blood and minimize DNA content while preserving extracellular proteins and tissue structure.

### 2.2. Histological Analysis

Both unprocessed and post-processed tissue allografts were fixed in 10% NBF, embedded in paraffin, sectioned, and stained with H&E and Masson’s trichrome using the established methods. This procedure was applied to 15 matched distinct donors of unprocessed adipose tissue and alloClae.

### 2.3. Scanning Electron Microscopy

Allograft samples were fixed in a 5 mL conical tube containing 4 mL of SEM fixative solution (4% formaldehyde/1% glutaraldehyde in PBS) and then rinsed with 0.1 M phosphate buffer before being placed in 1% Zetterquist’s osmium tetroxide for one hour. The samples were sequentially dehydrated in 70% ethanol for three cycles of 15 min, followed by 95% ethanol for three cycles of 15 min, and 100% ethanol for two cycles of 20 min. After dehydration, the samples were treated with hexamethyldisilazane (HMDS) for five minutes and allowed to air-dry in a desiccator. Once dried, they were mounted and sputter-coated with gold/palladium. Imaging was performed using a JEOL 6610 scanning electron microscope. Analysis was conducted on 7 distinct donors for alloClae.

### 2.4. Weight Compositional Analysis

Approximately 14 g of allografts were dehydrated in an oven dehydrator set at 98 °F for 9 h. Post-dehydration, weight loss was recorded for water content calculations. Allografts were then soaked in 99% isopropyl alcohol (IPA) overnight on an orbital shaker to extract lipids. After air-drying post-lipid extraction, the final weight was recorded for lipid content and connective tissue estimation. This procedure was applied to 14 allografts, each derived from a distinct donor.

### 2.5. Layer Compositional Analysis

A method adapted from [32] was used to quantify fat tissue, free oil, and aqueous layers. Approximately 14 g of allografts were transferred to pre-weighed conical tubes, and the total weight was recorded. Allografts were centrifuged at 3220× *g* for 3 min, after which the upper oil layer was removed and weighed. The percentage of each layer was calculated as the ratio of the weight of the designated layer to the total sample weight. This analysis was conducted on 32 allografts from 4 distinct donors.

### 2.6. DNA Quantification

For DNA quantitation, tissue (25 mg) was digested in a Proteinase K solution overnight at 55 °C, and DNA was isolated using a Zymo Quick-DNA Miniprep Plus Kit optimized for adipose DNA extraction. DNA was quantified using a NanoDrop. Analysis was conducted on 7 distinct donors for unprocessed adipose tissue and 11 distinct donors for processed alloClae allografts. At least 3 representative allografts were tested per donor in technical triplicates across two independent experiments. Lipid contamination during DNA extraction was reduced through optimized methodology. During optimization studies for lipid tissue extraction, care was taken to remove excess lipid after tissue digestion through centrifugation and pipetting of excess lipid from lysate. Extra care was taken to ensure low cross-contamination by using new micropipette tips after each step in the protocol. Lastly, the purity of the DNA samples was examined using two different NanoDrops to ensure the absence of contamination.

### 2.7. DAPI Staining of alloClae

Small sections of raw adipose tissue and alloClae (approximately 20–80 mg each) were prepared using sterile surgical scissors. For DNA visualization, tissue samples were transferred to individual microcentrifuge tubes containing 1 mL of PBS containing two drops of a NucBlue reagent (ThermoScientific Waltham, MA, USA, R37605), following the manufacturer’s protocol. The samples were then incubated at RT for 20 min with occasional agitation. After incubation, tissue sections were placed onto glass slides using sterile forceps, covered with a glass coverslip, and immediately imaged using a Cytation5 microscope at 10× magnification. Brightfield images were acquired, and fluorescent images of DNA were captured using a DAPI filter set (excitation/emission of 360/460 nm). Analysis was conducted on 3 distinct donors for unprocessed adipose tissue and alloClae.

### 2.8. Proteomic Analysis

Allografts for proteomic analysis were prepared and analyzed using the established methods [33]. In brief, alloClae was homogenized in 5% SDS in 50 mM triethylammonium bicarbonate and spun at 20,000× *g* for 15 min. The supernatant was collected and subjected to protein digestion with STrap. Protein peptides were pooled, dried in a vacufuge, resuspended in 5.0 mM acetic acid, and separated on nano-columns. After injections into a u-Precolumn Cartridge and dilutions with 0.1% formic acid, the samples were analyzed using nano-liquid chromatography–nanospray tandem mass spectrometry for protein identification. The proteome profile of alloClae was compared to matched raw tissue from 3 distinct donors to identify changes post-processing. We classified proteins by their functional categories using Gene Ontology (GO) analysis through Scaffold ver. 5.3.3 (Proteome Software, Inc., Portland, OR, USA).

### 2.9. Collagen Analysis

Collagen levels were determined using the QuickZyme Total Collagen Assay (QZBtotcol5), following the manufacturer’s instructions. The allograft (25 mg) was digested in 1 mL of 6 M HCl at 95 °C for 20 h. Post-digestion, the solution was centrifuged at 13,000× *g* for 10 min, and the supernatant was diluted to 4 M HCl. Collagen standards and allografts were mixed with an assay buffer, incubated at room temperature (RT) for 20 min, then treated with detection reagent, and incubated at 60 °C for 60 min with intermittent shaking. Absorbance was measured at 570 nm after cooling to RT. Analysis was conducted on 5 distinct donors of unprocessed tissue and 7 distinct donors with processed alloClae allografts. At least 3 representative allografts were tested per donor in technical triplicates across two independent experiments.

### 2.10. Elastin Quantification

Elastin content was assessed using a Fastin Elastin Assay Kit (BioColor, Belfast, UK, F4000), following the manufacturer’s protocol. Briefly, tissue (10–20 mg) was hydrolyzed in oxalic acid at 100 °C for 60 min. Allografts were centrifuged at 13,000× *g* for 10 min to collect the supernatant; then, elastin was precipitated and centrifuged again to discard the supernatant. The precipitate was dyed, mixed, and incubated before the final centrifugation to remove unbound dye. The bound dye was dissolved and quantified at 513 nm. Analysis was conducted on 6 distinct donors for unprocessed adipose tissue and 5 distinct donors for processed alloClae allografts. At least 3 representative allografts were tested per donor in technical triplicates across two independent experiments.

### 2.11. Laminin α4 ELISA

Allografts were homogenized using a BeadBlaster 24R Tissue Homogenizer in a RIPA buffer supplemented with Halt Protease Inhibitor, undergoing three cycles at 5 m/s for 30 s with 30 s rest intervals, followed by centrifugation at 6000× *g* for 15 min at 4 °C. Protein concentrations were determined using the BCA assay. The manufacturer’s ELISA instructions were followed (ThermoScientific, EH293RB). Optical density values were analyzed for interpolation with a second-degree polynomial line best fit to generate standard curves. Analysis was conducted on 10 distinct donors for unprocessed tissue and 13 distinct donors for processed alloClae allografts.

### 2.12. Biocompatibility Testing

L929 cells (ATCC, CCL-1) were seeded in a 24-well plate at densities of 30,000 cells per well, respectively. The cells were cultured in complete EMEM and incubated at 37 °C in a 5% CO_2_ atmosphere for 24 h. Allografts (0.2 mL) were transferred with forceps from a sterile container to the designated transwell insert. Control groups included cells cultured without allografts. After 72 h of incubation, assessments of viability, cytotoxicity, lipid peroxidation levels, and abundance were conducted on L929 cells. Cytotoxicity was evaluated using the CyQuant LDH assay (Invitrogen, Waltham, MA, USA, C20301), following the manufacturer’s instructions. Media (50 µL) from each well were incubated with a reaction buffer for 30 min at RT. Cytotoxicity percentages were calculated by comparing the LDH release in the experimental wells to the maximum LDH activity in the 1% Triton X-100 positive control. Cell viability was determined using the PrestoBlue HS Viability Assay (ThermoFisher Scientific, P50201), following the manufacturer’s instructions. Each well was incubated with PrestoBlue for one hour before fluorescence was measured at excitation/emission of 560/590 nm. Viability percentages were calculated relative to the control wells containing no graft. Lipid peroxidation levels were quantified using BODIPY 581/591 C11 (ThermoFisher Scientific, D3861). Each well was incubated with 5 µM of BODIPY at 37 °C for 30 min. The cells were washed, and fluorescence was read for reduced levels at excitation/emission of 581/591 nm and oxidized levels at 488/510 nm. The ratio of emission fluorescence intensities at 591 nm to 510 nm was used to estimate lipid peroxidation levels. Cellular abundance in L929 cells was measured by staining with crystal violet, washing, and dissolving in 1% SDS. After a 20 min incubation at RT, absorbance at 590 nm was measured to compare with the control cells. For these studies, 6 distinct donors of processed alloClae allografts were evaluated. At least 3 representative allografts were tested per donor in technical triplicates across two independent experiments.

### 2.13. Adipose-Derived Stem Cell (ASC) Adipogenesis Assay

ASCs (StemBioSys, San Antonio, TX, USA) were seeded at a density of 75,000 cells/well in 24-well plates cultured using an ADSC Growth Medium Bullet Kit (Lonza) and incubated at 37 °C with 5% CO_2_. After 24 h, allografts (0.2 mL) were added to designated transwell inserts, and ASC adipogenic differentiation was initiated. The positive control included cells without inserts grown in adipogenic induction medium (Lonza, Basel, Switzerland). The co-cultures were incubated for two weeks according to the manufacturer’s instructions. The cells were stained with Oil Red O and imaged at 3, 7, and 14 days to assess differentiation. The stain was then eluted using 100% IPA and quantified at 500 nm. This study evaluated 3 random allografts each from 8 distinct donors for processed alloClae allografts.

### 2.14. Adipose-Derived Stem Cell Attachment Assay

Allografts were transferred with forceps from a sterile container to a cloning ring placed at the base of a 24-well plate well. The tissue was evenly spread within the ring, followed by the addition of either growth media or 1 × 10^5^ ASCs. The allografts were incubated at 37 °C and 5% CO_2_ overnight. The samples were transferred to the 24-well plate, and 0.5 mL fresh media was added, with 0.1 mL fresh media added to each well every day. The allografts were collected after overnight incubation and fixed in a 5 mL conical tube containing 4 mL of SEM fixative solution (4% formaldehyde/1% glutaraldehyde in PBS).

### 2.15. Evaluation of alloClae in Athymic Mice

Female athymic nude mice (Foxn1, The Jackson Laboratories), 28-days-old upon arrival, were utilized for this study, aligning with previous studies using human adipose allograft [25,31]. Before the study began, mice were acclimated to the facility for 4 days, maintained on an ad libitum diet, and housed under a 12 h light/dark cycle. For each experimental group, n = 4–7 mice were tested at each designated evaluation timepoint (1, 3, and 6 months). Allografts (n = 3 donors) were prepared and transplanted utilizing an appropriately sized graft delivery tool. Under isoflurane-induced anesthesia, transplantation was performed by making a subcutaneous incision approximately 1–2 mm deep on the right side of the dorsum of the mouse, the transplants were evenly distributed under the skin, and incisions were sealed with Vetbond. Post-transplantation, the animals were monitored daily for any adverse effects. At predetermined timepoints, mice were euthanized in accordance with the AALAS guidelines. In vivo visual assessments were conducted based on criteria outlined in Supplementary Table S1. Following euthanasia, the transplants were extracted, fixed in 10% formalin, and sent to the University of Washington Histology and Imaging Core for analysis. Histological analysis was conducted by a DVM pathologist at the University of Washington and included H&E and Masson’s trichrome staining, as well as immunohistochemistry for mouse reactive perilipin-1 (Abcam Cambridge, UK, ab3526) and mouse specific CD-31 (Dianova, Hamburg, DE, DIA-310).

All animal procedures were approved by the Institutional Animal Care and Use Committee (IACUC) at Perret-Gentil Lab Animal Veterinary Services and carried out in accordance with the standards set forth by the American Association for Laboratory Animal Science (AALAS).

### 2.16. Statistical Analysis

All data were analyzed using GraphPad Prism version 10.2.3 (GraphPad Software, Boston, MA, USA). For quantifying DNA and ECM proteins, statistical differences were determined using Student’s two-tailed unpaired and paired *t*-tests for matched donor tissue and allografts. For in vitro biocompatibility analysis, an ordinary one-way ANOVA with Tukey’s post hoc test was used to determine significant differences between the groups. Significance was set at a 95% confidence level, with a *p*-value threshold of less than 0.05.

## 3. Results and Discussion

### 3.1. Tissue Structure and Composition of alloClae 

**Figure 1:**
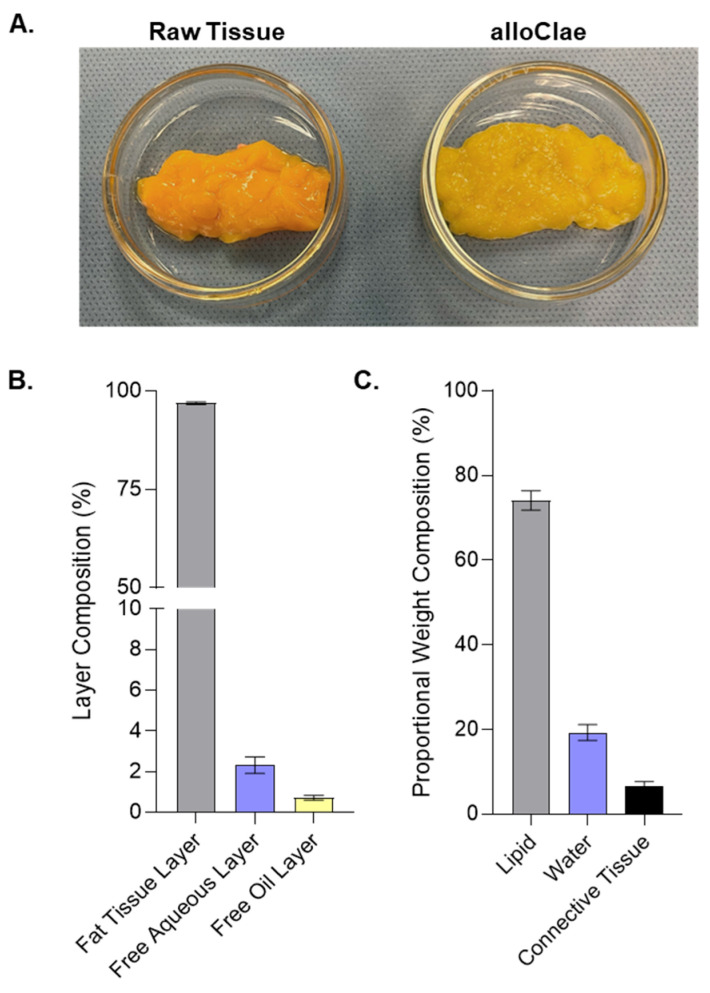
Physical appearance and compositional analysis of alloClae. (**A**) Representative images showcasing raw adipose tissue alongside the processed alloClae. (**B**) Layer composition of alloClae following centrifugation of 30 allografts. (**C**) Proportional weight composition of alloClae, based on analysis from 9 allografts, each derived from different donors. Data expressed as the means ± SEM.

Our study characterizes alloClae, a novel minimally processed adipose tissue allograft, optimized through a detergent-based protocol for soft tissue reconstruction. It undergoes a minimal series of processing steps that preserve not only the structural integrity but also the physical appearance of unprocessed adipose tissue (Figure 1A). This process enables the processing of up to 5 kg of adipose tissue at a time, with processing times of up to 14 h, representing a time efficiency improvement of over 100% compared to traditional decellularization protocols, which can involve multiple time steps extending up to 24 h each [19,22,23].

We conducted two distinct compositional analyses. First, we used a layer separation method following centrifugation to quantify the proportion of fat tissue, aqueous, and free oil layers. This approach is clinically relevant because excessive free oil in graft preparations leads to complications such as oil cyst formation, fat necrosis, and capsular contracture [34,35]. Our analysis showed that alloClae is composed of 93–99% fat tissue, 0–7% aqueous layer, and 0–2% free oil layers (Figure 1B). This composition contrasts favorably with reports indicating that fresh lipoaspirates processed via decantation, centrifugation, or commercial systems for fat autografting typically yield free oil layers of 2–9% [36], while another study observed lower free oil layer yields when using the current commercial protocols [37]. However, lipid composition in these studies may differ between processing methods, including differences in sample handling and the disruption caused by liposuction.

Second, we performed proportional weight compositional analysis, which estimates the internal makeup of the allograft in terms of water, lipids, and proteins. This analysis showed that alloClae contains approximately 15–36% water, 60–84% confined lipids, and 2–4% proteins (Figure 1C), aligning with the established benchmarks for native adipose tissue [38]. These relatively broad ranges observed for the lipid and water content reflect the inherent variability between donors. Collectively, these findings indicate that alloClae not only retains an internal composition resembling that of native adipose tissue, but also has a reduced free oil fraction, potentially mitigating graft-related complications.

**Figure 2:**
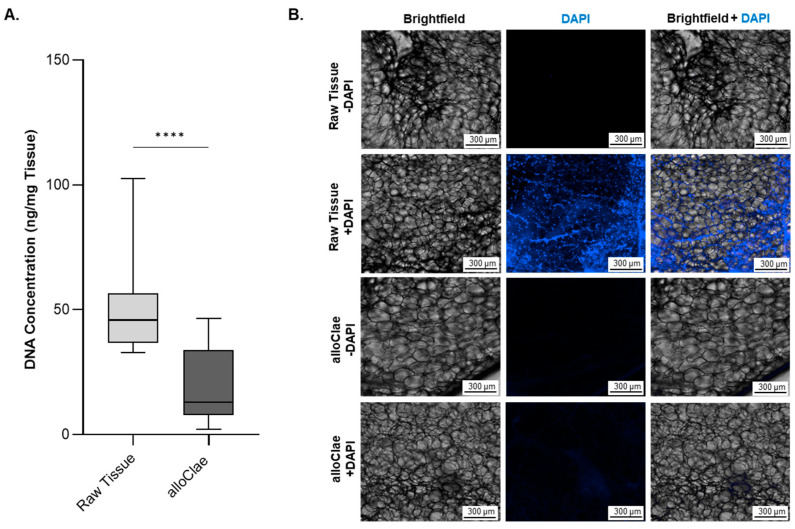
DNA content in raw adipose tissue versus alloClae. (**A**) DNA levels in raw unprocessed adipose tissue and alloClae. Data represent 7 distinct donors for unprocessed raw adipose tissue and 11 donors for alloClae, of which 5 donors are matched with unprocessed tissue. The results are presented as box whiskers showing the minimum and maximum points. Statistical differences between the raw tissue and alloClae were assessed using Student’s two-tailed unpaired test; **** *p* < 0.00001. (**B**) DAPI staining in raw adipose tissue and alloClae (10× magnification) showing nucleic acid content of raw tissue and post-processing. Distinct punctate nuclei can be observed in raw adipose tissue. Scale bar, 300 µm. Representative images of 3 donors.

To reduce the immunogenicity risk, alloClae underwent several washes to remove free lipids, cellular debris, and a final gamma irradiation step after the final packaging to reduce the DNA content. This process was aimed at ensuring minimal DNA presence, supported by both quantitative assessments and microscopic evaluation of tissue sections for nuclei presence. The results confirmed the minimization of DNA (Figure 2A) and nuclei (Figure 2B) content, aligning with the established criteria set by the scientific literature (less than 50 ng DNA/mg tissue) for immunogenicity risk mitigation [13,39]. While others observed differences in the DNA content in adipose tissue extractions, our findings align with the previously documented DNA levels in human adipose tissue and grafts derived from non-adipose tissues [40,41]. Fluorescent microscopy further supports reduced DNA levels in alloClae, as indicated by a decrease in DAPI fluorescence. Raw adipose tissue shows distinct punctate nuclei, while alloClae appears faint and diffuse (Figure 2B).

**Figure 3:**
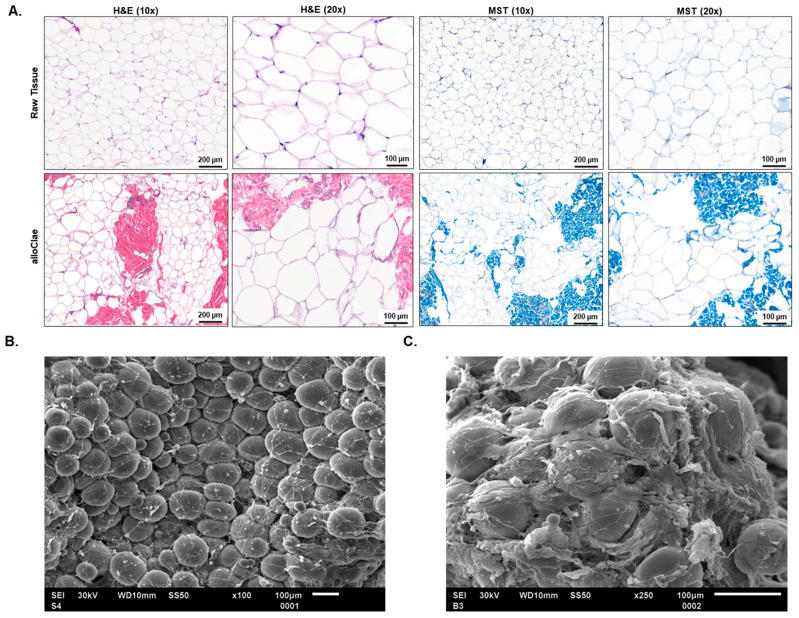
Structural analysis of adipose tissue in alloClae. (**A**) Comparison of the adipose tissue structure before (raw) and after (alloClae) processing. Tissue cross-sections were stained using hematoxylin and eosin (H&E) and Masson’s trichrome (MST) to highlight structural changes post-processing. Images were selected from 15 matched donors, examining at least five fields of view on each histological cross-section. Scale bar, 200 µm (10× magnification) and 100 µm (20× magnification). (**B**,**C**) Scanning electron microscopy of alloClae at 100× (**B**) and 250×. Images were selected from 7 distinct donors. (**C**) Magnification. Scale bar, 100 µm.

Histological evaluations post-processing revealed that alloClae retained its native honeycomb adipose tissue structure, reflecting minimal manipulation of the tissue’s structural characteristics (Figure 3A), which is vital for the graft’s structural integrity. Masson’s trichrome staining highlights the enrichment of collagen, stained blue, suggesting the preservation of a three-dimensional structural matrix essential for supporting healthy tissue integration and growth (Figure 3A). Preservation of structural integrity is further supported by scanning electron microscopy, which demonstrated that the adipose tissue structure remained fully intact, preserving the characteristic round shape (Figure 3B). Additionally, the presence of the extracellular matrix was observed at a higher magnification (Figure 3C).

### 3.2. Proteomic and Quantitative Analysis of Extracellular Matrix Proteins in alloClae

**Figure 4:**
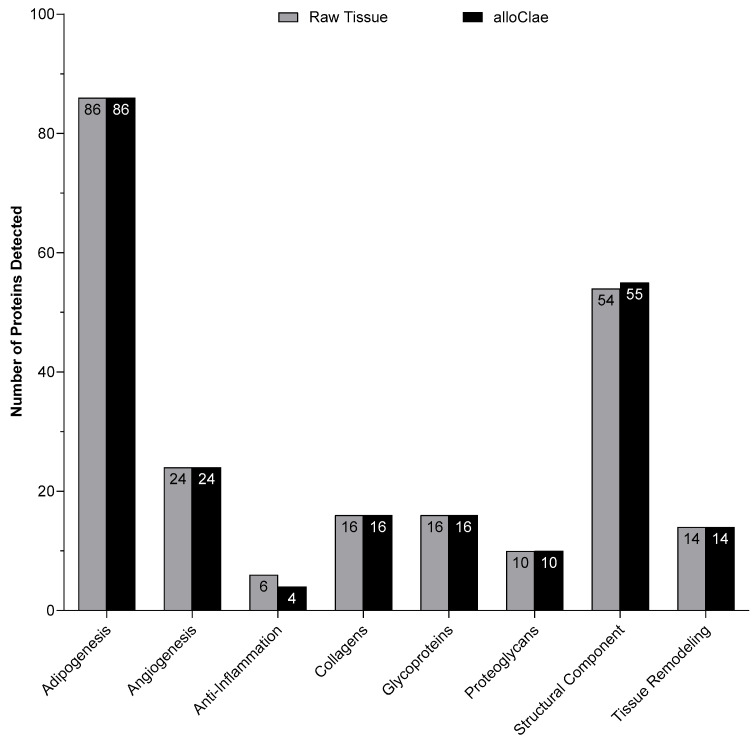
Comparison of the number of proteins detected in each category between matched raw adipose tissue and alloClae.

Adipose tissue engineering relies on grafts that provide a favorable tissue microenvironment, and a key requirement for successful adipose tissue engineering is to preserve proteins that support host cellular activities such as angiogenesis, adipogenesis, anti-inflammatory, tissue remodeling, and extracellular matrix (ECM) components (collagens, glycoproteins, proteoglycans, and structural). To determine whether these critical proteins remain present following tissue processing, we performed a proteomic analysis comparing alloClae with matched raw donor tissue. We used Gene Ontology (GO) analysis through Scaffold (Proteome Software, Inc., USA) to classify proteins by their functional categories. Together, the functional categories had 226 specified proteins, and proteomic results showed that 223 out of 226 of these functional proteins were retained in alloClae compared to native adipose tissue (Figure 4). Out of the 54 ECM’s structural proteins identified, only one was absent in alloClae, whereas 4 out of the 6 anti-inflammatory-associated proteins were retained in alloClae (Table 1). Furthermore, two known ECM’s structural proteins were identified in the alloClae samples in contrast to the raw adipose samples as a result of the assay detection threshold and the sample’s protein concentration (Table 1). Overall, these findings indicate that the detergent-based protocol used for alloClae preserves a broad range of proteins essential for angiogenesis, adipogenesis, anti-inflammatory activity, ECM components, and tissue remodeling, which are factors that are critical for graft integration within the host and long-term graft stability [24,42,43].

**Figure 5:**
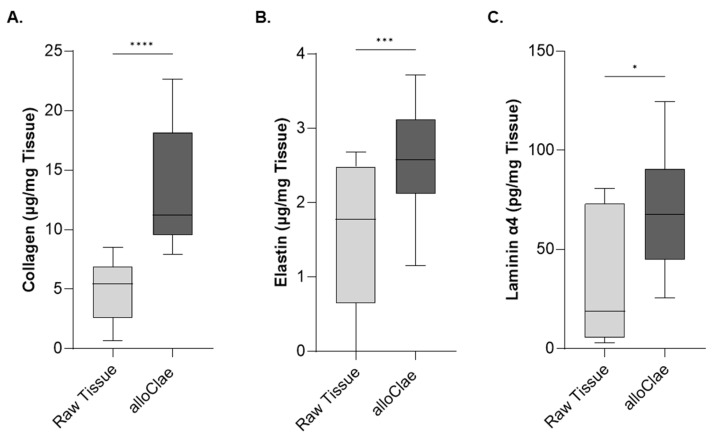
Extracellular matrix protein analysis in raw adipose tissue and alloClae. (**A**) Analysis of collagen levels in raw adipose tissue and alloClae. Representative data from 5 donors for raw tissue and 7 donors for alloClae. Analysis conducted in 3 allografts per donor in technical triplicates across two independent experiments. (**B**) Elastin levels in 6 donors of raw adipose tissue and 5 donors of alloClae. Analysis conducted in 3 allografts per donor in technical triplicates across two independent experiments. (**C**) Levels of laminin α4 across 10 donors of raw tissue and 13 donors of alloClae. The results are presented as box whiskers showing the minimum and maximum points. Statistical differences assessed using Student’s two-tailed unpaired and paired *t*-tests for matched samples; * *p* < 0.01; *** *p* < 0.0001; **** *p* < 0.00001.

Building on these findings, we conducted a targeted quantitative analysis of ECM proteins that have been shown to form the structural framework for host cell attachment, migration, and differentiation [44]. Structural image analyses showed that the ECM remained intact in the grafts (Figure 3), prompting us to measure levels of collagens, elastins, and laminin α4, each contributing to tissue architecture and cellular support. Quantification of these proteins revealed that the collagen content was 1.5 times more concentrated in alloClae (Figure 5A). Collagen is the major structural component of the ECM, providing the framework for the attachment of other proteins such as laminin [45]. The increase in collagen is notable, as collagen ensures the structural integrity of the graft, supporting cell adhesion and graft stability. Similarly, total elastins showed a 0.5-fold concentration (Figure 5B). The presence of elastins in alloClae suggests the retention of elasticity to withstand deformation [46]. Laminin α4 levels were also 1.5 times higher following the tissue processing protocol (Figure 5C). Laminins mediate cell interactions, attachment, and migration, all of which are important for the successful integration of alloClae within the host tissue [47,48]. Furthermore, laminins have been shown to support vascular growth, further facilitating tissue remodeling and stabilization [49]. The enhanced laminin levels in alloClae have the potential to support cell–ECM interactions, aiding in the anchoring of cells to the collagen framework, further stabilizing the allograft.

In contrast, fully decellularized adipose allograft (dAA) products often face a significant reduction or complete loss of these key ECM components due to harsh protocols involving mechanical manipulation, enzymatic digestion, chemical extraction methods, and extreme temperature conditions [24,50]. However, our proteomic and ECM quantitative analyses demonstrate that alloClae retains these essential proteins within the graft. Coupled with structural analyses (Figure 3), these findings suggest that alloClae preserves the ECM integrity needed for successful graft–host integration and long-term stability.

### 3.3. In Vitro Biocompatibility of alloClae

**Figure 6:**
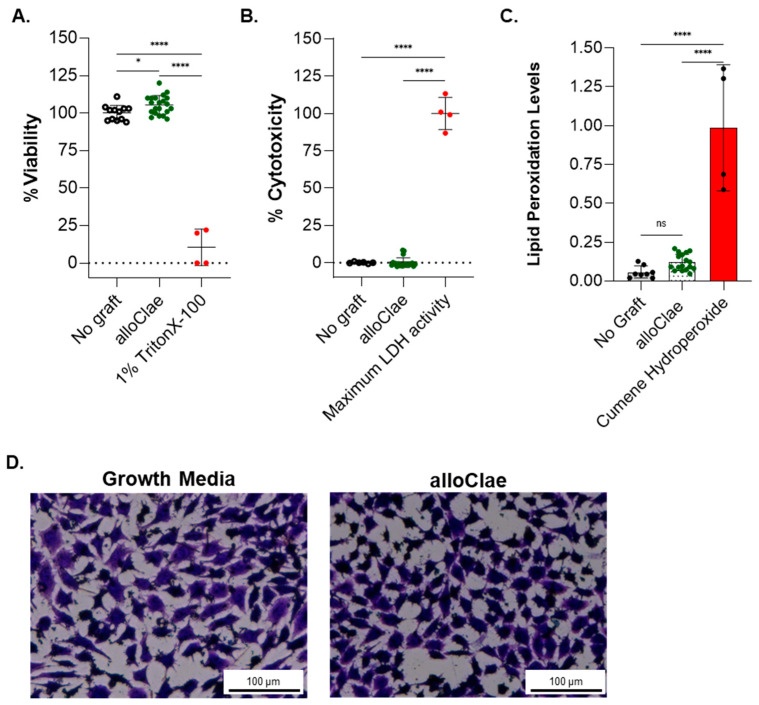
In vitro biocompatibility of alloClae. (**A**) Viability, (**B**) cytotoxicity, and (**C**) lipid peroxidation assessment in L929 cells after 72 h of co-culture with either growth media only or alloClae. Lipid peroxidation was detected using a BODIPY 581/591 C11 probe, which fluoresces green in the presence of non-oxidized lipids and shifts to red fluorescence upon lipid peroxidation. Quantification of the fluorescence intensity ratio (red/green) was used as an indicator of lipid peroxidation. Positive control for lipid peroxidation was cumene hydroperoxide. (**D**) Crystal violet staining of viable L929 cells. Brightfield images taken at 4× magnification. Scale bar, 100 µm. All tests were performed in technical triplicates across two independent experiments, with quantitative data expressed as the means ± SD. Statistical differences were assessed using one-way ANOVA with Tukey’s post hoc test; * *p* < 0.01; **** *p* < 0.00001. ns = non-significant.

To evaluate alloClae’s biocompatibility and its potential impact on tissue integration, we employed a multiplex approach combining cytotoxicity and cell viability assays to assess the biocompatibility of alloClae in L929 fibroblast cells. The PrestoBlue viability assay, which measures metabolic activity, indicated that cell viability remains unchanged (Figure 6A). Additionally, the lactate dehydrogenase (LDH) assay was performed to assess potential cytotoxicity by comparing the LDH release in alloClae-treated cells to the maximum LDH release observed in Triton X-100-treated L929 cells (positive control). The results indicate that alloClae does not induce cytotoxic effects, as supported by LDH release levels comparable to those of the negative control (Figure 6B). To further evaluate cellular responses to alloClae, we assessed lipid peroxidation levels using the C-11 BODIPY assay. Lipid peroxidation is a marker of oxidative stress that can result in membrane damage and impaired cell function. The results confirm that alloClae does not induce lipid peroxidation, indicating that its presence does not contribute to oxidative stress in L929 cells (Figure 6C). Furthermore, the crystal violet staining assay used to measure viability [51] confirmed no adverse effects on cell density or cell morphology of L929 cells (Figure 6D).

### 3.4. Adipose-Derived Stem Cell Attachment and Differentiation with alloClae

**Figure 7:**
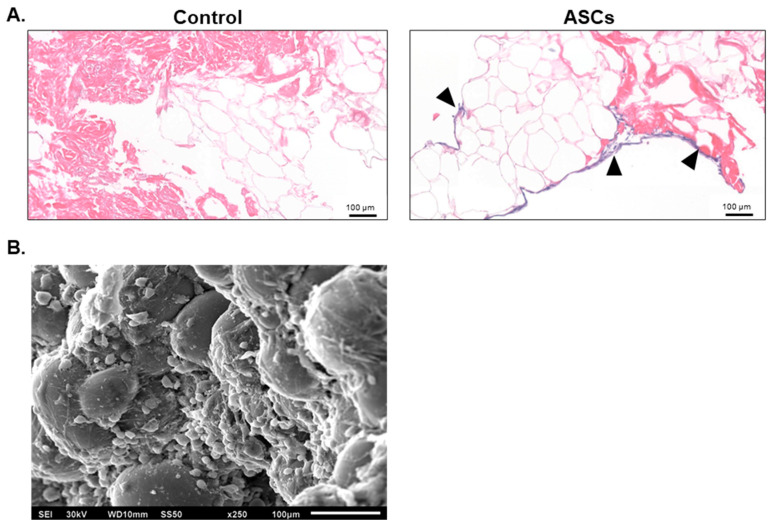
Attachment of adipose-derived stem cells to alloClae. (**A**) Hematoxylin and eosin histology scans showing cell attachment and infiltration following 3 days of incubation with alloClae. Black triangles highlight the presence of nuclei. Histology scale bar, 100 µm. (**B**) Scanning electron microscope image (250× magnification) showing cell attachment following 3 days of incubation on alloClae. SEM scale bar, 100 µm.

The retention of native ECM proteins is recognized to provide a stable structural framework for cellular adhesion, migration, and proliferation [24,36]. Following confirmation of alloClae’s biocompatibility, we investigated the graft’s capacity to support cell attachment, focusing on human adipose-derived stem cells (ASCs), which are well-known for their multipotent properties [43]. ASCs were seeded onto alloClae grafts for three days under in vivo culture conditions; after three days, alloClae was fixed and stained with H&E or processed for SEM. H&E staining and SEM scans revealed ASC attachment at the periphery of alloClae (Figure 7). These results indicate that alloClae supports ASC attachment. Further, alloClae was compared to a commercially available dAA, a commonly used adipose tissue graft obtained through different decellularization processing methods. In this comparison study, the results suggest alloClae displays comparable levels of ASC attachment (Supplementary Figure S1). Notably, alloClae achieves this without undergoing a complete decellularization process.

**Figure 8:**
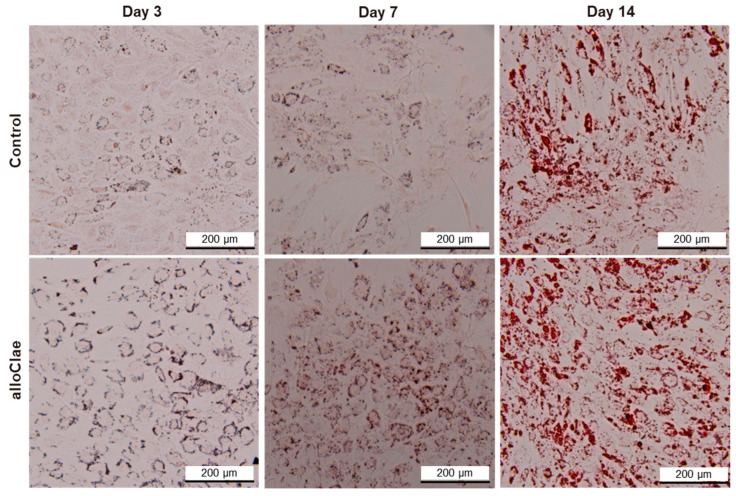
Adipose-derived stem cell differentiation with alloClae. ASCs stained with Oil Red O to visualize lipid droplets. Phase-contrast images (4× magnification) of the ASCs that underwent adipogenic differentiation for 3, 7, and 14 days. The cells were grown in a differentiation medium in the absence of a graft (control, n = 3 per timepoint) and 5 donors of alloClae (n = 15 samples per timepoint). Scale bar, 200 µm.

A key objective in adipose tissue engineering is to ensure that host cells not only attach and survive, but also differentiate into mature adipocytes capable of replacing graft fat cells. To determine whether alloClae provides a conducive environment for stem cell differentiation, ASCs were cultured in the presence of alloClae under adipogenic conditions over 14 days. Oil Red O staining was employed to detect lipid droplets, a hallmark of adipogenic differentiation. Morphological changes indicative of differentiation are observed as early as on day 3 in cells treated with alloClae. By this time, faint lipid droplet formation is detected in both control and alloClae-treated cells, as indicated by Oil Red O staining, which is weak in the control-treated cells (Figure 8). After 7 days of culture, lipid droplet accumulation became more pronounced in the cells exposed to alloClae compared to the control-treated cells. By day 14, cells treated with alloClae predominantly differentiate into cells characterized by a significant lipid droplet accumulation, as determined by Oil Red O staining (Figure 8).

These in vitro findings demonstrate that alloClae supports ASC attachment and differentiation, both of which are essential for graft integration and stability. The observed differentiation of ASCs suggests that alloClae provides an environment conducive to the formation of new adipocytes, which may replace graft components with host-derived cells over time. This aligns with mechanisms of graft remodeling that rely on infiltrating stem cells to support tissue reconstruction and functionality [50]. While these in vitro findings highlight alloClae’s potential, they do not represent the complexity of in vivo environments. Therefore, animal studies were conducted to evaluate alloClae’s potential for soft tissue reconstruction and repair.

### 3.5. Long-Term Integration and Stability of alloClae in an Athymic Mouse Model

A 6-month longitudinal study was conducted to evaluate overall tissue integration and the retention and stability of alloClae transplants in an athymic mouse model. Athymic nude mice, the gold standard for xenotransplantation research in fat grafting, particularly with human-derived grafts such as alloClae, were selected for this study [30,52]. These mice lack T cells but retain other immune components such as B cells and macrophages, which help to minimize immune rejection of human-derived grafts while enabling clear observations of graft–host interactions and facilitating a partial understanding of alloClae’s biocompatibility [52,53,54,55]. Additionally, their hairless nature allows for visual tracking of these grafts over time, which underscores their suitability for assessing alloClae retention and integration.

**Figure 9:**
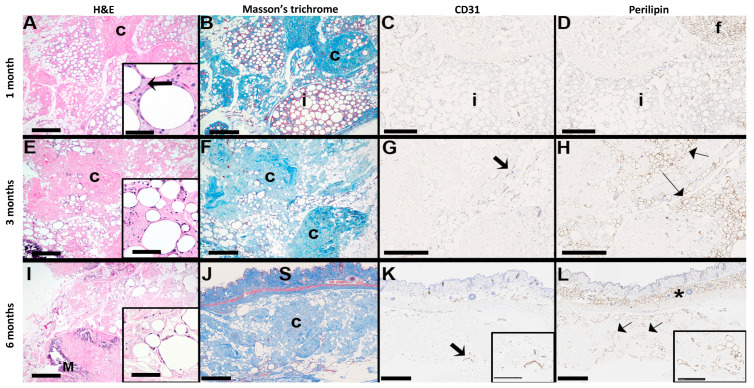
Histopathological assessment of alloClae transplants in athymic mice. Histology scans of H&E, Masson’s trichrome, mouse reactive anti-perilipin, and mouse specific anti-CD31 stains of an alloClae adipose tissue graft transplanted subcutaneously in athymic mice. The arrow in the inset for (**A**) denotes a foamy macrophage; c = collagen (blue on Masson’s trichrome); f = mouse adipose tissue in the upper right corner of (**D**); adipocytes within the implant (i) do not label for perilipin. In (**G**,**K**), thick arrows indicate positively stained vessels. In (**I**), M = focus of mineralization. In (**H**,**L**), thin arrows indicate positively stained adipocytes. (**J**–**L**) Orientation of the implant in relation to the mouse skin (S). The asterisk in (**L**) shows the normal subcutaneous fat. (**A**,**B**,**E**,**F**,**H**–**L**) Bar = 500 µm. Inset (**A**), bar = 50 µm; inset (**E**,**I**), bar = 100 µm; (**C**,**D**,**G**) bar = 250 µm; inset (**J**,**K**), bar = 250 µm.

Integration was evaluated through both visual and histopathological assessments at 1-, 3-, and 6-month post-operative intervals. Early qualitative assessments of the transplants provide evidence for integration, employing a scoring system to assess transplant integration with overlying skin and underlying tissue, coloration of the transplant, and vascularization (Supplementary Table S1). At one-month post-transplantation, visual inspection scores indicated initial stages of integration of the transplant and initial signs of vascularization, complemented by minimal coloration changes (Table 2). A mild to occasionally moderate inflammatory response was observed, characterized by a multifocal presence of foamy macrophages with minimal neutrophil presence (Figure 9). This initial inflammatory response is necessary to initiate remodeling of the tissue for transplant integration [56,57].

Three months into the study, visual assessment scores noted improvements in skin integration and vascularization (Table 2). Histological examination showed reduced immune cell infiltration and retention of the adipocyte structural appearance in an increased fibrous matrix composed of alloClae’s retained ECM components over time in the host (Figure 9) [10,24,26,58]. The examination of the fibrous matrix also showed cell adhesion, blood supply, and adipogenesis, all of which are essential for graft stability [59]. The transplant site did not exhibit a fibrous capsule, significant inflammation, or large cystic necrosis and degeneration.

Immunohistochemistry was performed using mouse reactive anti-perilipin-1 and anti-CD31 antibodies to assess host-specific adipogenesis [60] and new blood vessel formation [61], respectively (Figure 9). This analysis confirmed that newly formed adipose tissue and blood vessels in the graft originated from the host rather than the human-derived alloClae. Significant cell infiltration into the graft tissue, mainly at the peripheral regions, was observed, suggesting successful integration with the surrounding tissue. These observations are consistent with previous studies indicating that host cell recruitment plays a vital role in long-term graft stabilization and regeneration [40,43,52].

Evaluations at six months assessed whether these remodeling processes progressed, plateaued, or stabilized. Visual assessment scores for underlying tissue integration and vascularization remained consistent (Table 2). Histological evaluations did not show further significant progression in adipogenesis and angiogenesis, suggesting a potential stabilization phase (Figure 9). Beyond three months, both adipogenesis and angiogenesis began to plateau, suggesting the primary window for tissue replacement may be within these first three months. This observation is indicative of alloClae’s integration and stabilization within the host tissue, as demonstrated by cell infiltration and transplant attachment to host tissue (Figure 9). Importantly, the transplants did not migrate and were consistently retained at the transplantation site over 6 months.

Notably, no oil cysts or host tissue necrosis were observed at any timepoint, backing the conclusion that alloClae supports balanced remodeling within the graft in athymic nude mice and reduces complications typically associated with fat grafting, such as ischemia or localized necrosis [62]. Our findings suggest that in this animal model, alloClae provides a conducive microenvironment for cell attachment, differentiation, and tissue regeneration, highlighting its potential as a solution for soft tissue reconstruction.

Altogether, these findings support the potential of alloClae as a viable alternative to autologous fat grafting. Additional studies are warranted to quantify the long-term volume retention. Although these studies were conducted in an animal model, which may not fully reflect the complexity of human host–graft interactions, they establish a strong foundation for future translational research.

## 4. Conclusions

The development of alloClae represents a significant advancement in tissue engineering for soft tissue reconstruction. By overcoming key limitations such as extensive processing periods and protein loss associated with the existing graft materials and techniques, alloClae offers a promising new avenue for restoring form, cushioning, and support following significant tissue volume loss. Importantly, alloClae is off-the-shelf-ready for clinical use, offering a significant advantage in terms of convenience for soft tissue reconstruction applications. The outcomes of this study further support alloClae’s potential for clinical applications in soft tissue reconstruction, emphasizing its positioning as a viable option for clinicians and patients.

## 5. Patents

The subject matter of this manuscript is patent-pending and the intellectual property of BioCreations Medical, LLC.

## Figures and Tables

**Table 1:** Proteins detected in raw adipose tissue and alloClae grouped by functional roles.

Functional Role	Related Proteins Detected in Raw Tissue and alloClae	Related Proteins Detected Only in Raw Tissue	Related Proteins Detected Only in alloClae
Glycoproteins	LMNB1, LMNB2, LAMA2, LAMA3, LAMA4, LAMA5, LAMB1, LAMB2, LAMC1, EMILIN1, EMILIN2, FN1, FBN1, MYOC, THBS1, VIT, VWF, ECM1		
Collagens	COL1A1, COL3A1, COL4A1, COL5A1, COL6A1, COL12A1, COL14A1, COL15A1, COL18A1, COL28A1, COL1A2, COL4A2, COL5A2, COL6A2, COL6A3, COL6A6		
Proteoglycans	ASPN, BGN, DCN, HSPG2, LUM, OGN, PRELP, VCAN, FMOD, AGRN		
ECM’s structural components	SERPINA1, LGALS1, PODN, MFAP4, VWF, VCAN, LTBP1, SPARCL1, ADAMTSL4, EFEMP1, EFEMP2, FBLN5, LTBP2, OGN, PRELP, LUM, TNXB, ECM1, COMP, MMP9, APOE, LGALS3BP, ANGPTL4, LPL, ASPN, BGN, ADAMTS15, TGM2, MYOC, VWA1, FBN1, TNC, TGFBI, COL1A1, COL3A1, COL4A1, COL5A1, COL6A1, COL12A1, COL14A1, COL15A1, COL18A1, COL28A1, COL1A2, COL4A2, COL5A2, COL6A2, COL6A3, COL6A6, SPARC, LOX1, CALR, ANXA2	CCDC80	ADAMTS15, HMCN2
Angiogenesis	HMCN2, SPARC, PXDN, LOXL2, COL18A1, COL4A2, COL4A1, COL5A1, COL28A1, COL6A1, COL15A1, ANXA2, EFEMP2, FBLN4, THBS4, LAMB2, VWA1, FBN1, TNC, AMBP, ARP4, ADAMTSL4, FMOD, CASP		
Anti-inflammatory activity	TGF-βR2, TGF-βR3, CD40, TNFSF13	SMAD5, STAT5A	
Tissue remodeling	PDGFRα, PDGFRβ, TGF-βR2, TGF-βR3, FGF-2, GRN, MATN2, TIMP-1, TIMP-2, TIMP-3, MMP2, MMP9, ADAMTS4, ADAMTS15		
Adipogenesis	MYMX, AAAS, ABHD15, ACAT1, ACOX1, ACSL1, ACVR1C, ADAMTS15, ADAMTS4, ADGRF5,ADIPOQ, ADIRF, AGPAT2, AKR1C2, AKT1, ANP32E, APOE, ARHGEF15, ASPH, ATG7, C3, CAV1, CAV2, CD248, CD5L, CLDN5, CLMP, COL6A1, COMP, CTBP1, DARS1, DHRS7B, DOCK11, DSG2, DSP, ECHDC3, ENO1, ENPP2, FAP, FASN, FBN1, FTO, FZD4, GPD1, GPT2, GRN, HEPACAM, HEXA, HSPB7, IGF1R, INSR, JUP, LIPA, LMNA, LOXL2, LPL, LRP1, MFN2, MTARC1, OLFML3, PARP1, PGRMC2, PIK3CA, PIK3R1, PLAAT3, PLXNA4, PNPLA2, AMPK1, PRKAR2B, RARRES2, RHOA, RUVBL1, S100B, SCARA5, SLC25A25, SLC27A4, SNAPIN, SNX17, SPART, SPTLC2, SRPX2, SVEP1, TBL1XR1, TPP1, TRPM4, VIM, VIT		

**Table 2:** In vivo transplant assessment.

	Integration to the Overlying Skin (0–3)	Integration to the Underlying Tissue (0–3)	Coloration of the Transplant (0–4)	Vascularization (0–4)
1 month	1.86	1.86	0.71	1.86
3 months	2.00	1.67	1.00	2.00
6 months	2.00	2.00	1.00	2

## Data Availability

The data presented in this study are available upon request from the corresponding author.

## Supplementary Materials

The following supporting information can be downloaded at: https://www.mdpi.com/article/10.3390/bioengineering12060612/s1, Supplementary Figure S1: Adipose-derived stem cell attachment to alloClae and decellularized adipose allograft (dAA) after 5 days. Hematoxylin and eosin histology scans (20× magnification) showing cell attachment and infiltration following 5 days of incubation in alloClae and commercially available dAA. Black triangles highlight the presence of nuclei. Histology scale bar, 100 µm; Supplementary Table S1: Transplant observations evaluation criteria.

## Abbreviations

The following abbreviations are used in this manuscript:

AALAS	American Association for Laboratory Animal Science
AATB	American Association of Tissue Banks
ASC	Adipose-derived stem cell
CFR	Code of Federal Regulations
dAA	Decellularized adipose allograft
DNA	Deoxyribonucleic acid
ECM	Extracellular matrix
FDA	Food and Drug Administration
FOV	Field of view
GO	Gene Ontology
H&E	Hematoxylin and eosin
HMDS	Hexamethyldisilazane
IACUC	Institutional Animal Care and Use Committee
LDH	Lactate dehydrogenase
MST	Masson’s trichrome
SD	Standard deviation
SEM	Standard error of mean
