# Supplementary material for: Advancing Soft Tissue Reconstruction with a Ready-to-Use Human Adipose Allograft
Source: Bioengineering (Basel). 2025 Jun 4;12(6):612. doi: 10.3390/bioengineering12060612 (PMC12189649; doi:10.3390/bioengineering12060612)
Supplement: Supplementary file 1 [file bioengineering-12-00612-s001.zip › bioengineering-3429593-supplementary.pdf]

Supplementary Materials

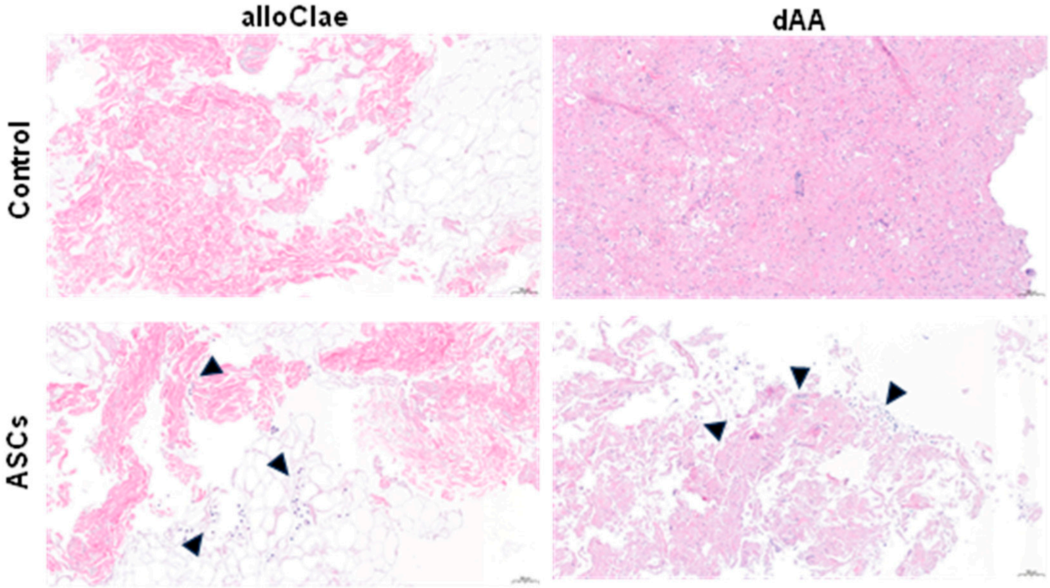

Supplmental Figure S1. Adipose derived stem cell attachment to alloClae and decellularized adipose allograft (dAA) after 5 days. Hematoxylin and Eosin histology scans (20× magnification) showing cell attachment and infiltration following 5 days incubation in alloClae and commerically available dAA. Black triangles highlight the presence of nuclei. Histology scale bar 100 µm.

Supplemental Table S1. Transplant Observations Evaluation Criteria

**Transplant integration to overlying skin**	**Qualitative assessment of transplant integration into overlying skin.**<br>0 = Transplant appear free from integration into overlying skin<br>1 = Transplant appears slightly integrated (1–33%) into overlying skin<br>2 = Transplant appears moderately integrated (33–66%) into overlying skin<br>3 = Transplant appears securely integrated (>66%) into overlying skin
**Transplant integration to underlying tissue**	**Qualitative assessment of transplant integration into underlying tissue.**<br>0 = Transplant appear free from integration into underlying tissue<br>1 = Transplant appears slightly integrated (1–33%) into underlying tissue<br>2 = Transplant appears moderately integrated (33–66%) into underlying tissue<br>3 = Transplant appears securely integrated (>66%) into underlying tissue
**Coloration of the Transplant**	**Qualitative assessment of overall transplant coloration; notation of "speckling" is included with each level below.**<br>0 = the majority (>75%) Transplant color appears white, off-white or yellow, similar to original Transplant coloration<br>1 = the majority (>75%) Transplant color appears beige<br>2 = the majority (>75%) Transplant color appears brown<br>3 = the majority (>75%) Transplant color appears dark brown to black<br>4 = the majority (>75%) Transplant color black

**Vascularization**	**Qualitative assessment of vascularization of transplant after excision evaluating superior and inferior aspects. If any blood vessel exhibits branching patterns, any branching vessel that is more than 1/2 the length of the primary vessel which it branches off will be counted as a new vessel. If the branching vessels are less than 1/2 the length of the primary vessel, they will be counted as part of the primary vessel. Blood vessel length will be measure using digital Vernier calipers and the length is recorded.**<br>0 = there are no visible blood vessels present on the Transplant surface after excision<br>1 = slight vascularization of between 1–3 clearly visible individual vessels within Transplant surface after excision<br>2 = moderate vascularization of between 4–6 clearly visible individual vessels within Transplant surface after excision<br>3 = intermediate vascularization of between 7–9 clearly visible individual vessels within Transplant surface after excision<br>4 = high vascularization of greater than 9 clearly visible individual vessels within the Transplant surface after excision
